# *S*-allylmercaptocysteine inhibits TLR4-mediated inflammation through enhanced formation of inhibitory MyD88 splice variant in mammary epithelial cells

**DOI:** 10.1038/s41598-024-81304-2

**Published:** 2024-11-28

**Authors:** Miyuki Takashima, Masahiro Kurita, Haruhi Terai, Feng-Qi Zhao, Jun-ichiro Suzuki

**Affiliations:** 1 Drug Discovery Laboratory, Wakunaga Pharmaceutical Co., Ltd, 1624, Koda-cho, Akitakata-shi, Hiroshima 739-1195 Japan; 2 Central Research Institute, Wakunaga Pharmaceutical Co., Ltd, 1624, Koda-cho, Akitakata-shi, Hiroshima 739-1195 Japan; 3 Department of Animal and Veterinary Sciences, University of Vermont, 102 Terrill, 570 Main Street, Burlington, VT 05405 USA

**Keywords:** Cell biology, Immunology, Molecular biology, Molecular medicine

## Abstract

Mastitis is an inflammatory disease affecting mammary tissues caused by bacterial infection that negatively affects milk quality and quantity. *S*-Allylmercaptocysteine (SAMC), a sulfur compound in aged garlic extract (AGE), suppresses lipopolysaccharide (LPS)-induced inflammation in mouse models and cell cultures. However, the mechanisms underlying this anti-inflammatory effect remain unclear. In this study, we demonstrated that oral administration of AGE suppressed the LPS-induced immune response in a mastitis mouse model and that SAMC inhibited LPS-induced interleukin-6 production and nuclear factor κB p65 subunit activation in HC11 mammary epithelial cells. Global phosphoproteomic analysis revealed that SAMC treatment downregulated 910 of the 1,304 phosphorylation sites upregulated by LPS stimulation in mammary cells, including those associated with toll-like receptor 4 (TLR4) signaling. Additionally, SAMC decreased the phosphorylation of 26 proteins involved in pre-mRNA splicing, particularly the U2 small nuclear ribonucleoprotein complex. Furthermore, we found that SAMC increased the production of the myeloid differentiation factor 88 short form (MyD88-S), an alternatively spliced form of MyD88 that negatively regulates TLR4 signaling. These findings suggest that SAMC inhibits TLR4-mediated inflammation via alternative pre-mRNA splicing, thus promoting MyD88-S production in mammary epithelial cells. Therefore, SAMC may alleviate various inflammatory diseases, such as mastitis, by modulating immune responses.

**Supplementary Information:**

The online version contains supplementary material available at 10.1038/s41598-024-81304-2.

## Introduction

Mastitis is an inflammation of the mammary gland caused by an intramammary infection with pathogenic bacteria, such as *Escherichia coli* (*E. coli*) and *Staphylococcus aureus*, in mammals, including cows, dogs, and humans^1–3^. It causes considerable economic losses, especially for dairy cows, primarily because of a decline in both the quality and quantity of milk production^4,5^. Epithelial cells in the mammary gland play important roles not only in milk production^6^ but also as the first line of defense against pathogens^7,8^. Mammary epithelial cells (MECs) recognize bacterial components through pattern recognition receptors, including toll-like receptors (TLRs), and produce pro-inflammatory cytokines, such as interleukin 6 (IL-6), tumor necrosis factor α (TNFα), and IL-1β, as well as the chemokines C-X-C motif chemokine ligand 1 (CXCL1) and C-C motif chemokine ligand 2 (CCL2)^9^, which recruit other immune cells to the infection sites to ultimately eliminate bacteria^10,11^. However, long-term inflammation can lead to the damage and dysfunction of both MECs and surrounding tissues, thereby reducing milk production^8,12^.

TLRs recognize microbe-associated molecular patterns (MAMPs) such as lipopolysaccharide (LPS), lipoteichoic acids, and flagellin^11,13^, thus acting as pathogen sensors. TLR4, a transmembrane protein expressed on the surface of various cell types, including epithelial and immune cells, is activated by LPS present in the outer membrane of gram-negative bacteria^14–16^, such as *E. coli*. TLR4 polymorphisms have been associated with resistance to mastitis and improve the milk yield and quality^17,18^, likely due to a reduced interaction between TLR4 and its adaptor proteins. These findings indicate that TLR4 plays a significant role in the exacerbation of mastitis. The binding of a ligand to TLR4 promotes the formation of TLR4 dimers. This in turn facilitates the association of myeloid differentiation response protein 88 (MyD88), a common adaptor protein of TLRs, with the cytoplasmic toll/IL1R domain of TLRs, as well as the oligomerization of MyD88 through interactions with itself and self-assembly via its death domain^19^. Subsequently, the MyD88 oligomer recruits IL-1 receptor-associated kinase 4 (IRAK4) and IRAK2 to form a myddosome^20^. This complex subsequently activates nuclear factor-κB (NF-κB) and induces the production of pro-inflammatory cytokines^21^. TLR4 signaling flux depends on the size and number of myddosomes^22,23^. In addition, damage-associated molecular patterns (DAMPs) released from dead cells, such as chromatin-associated high-mobility group box 1 (HMGB1), heat shock protein 70 (HSP70), S100 proteins, ATP, host DNA, and RNA, can also activate TLRs^24^. Indeed, in bovine mastitis, HMGB1 and HSP70 levels in milk are elevated and the TLR4 signaling pathway is activated^12,25,26^. Thus, both MAMPs and DAMPs may accelerate the inflammatory positive feedback loop by activating TLRs in the mammary glands^13^. Moreover, inhibiting TLR4 signaling attenuated inflammation in the mammary gland and protected mammary tissues in an LPS-induced murine mastitis model^27,28^, suggesting that effectively modulating TLR4 signaling is crucial for treating mastitis.

Aged garlic extract (AGE) is prepared by aging raw garlic (*Allium sativum* L.) in aqueous ethanol for over 10 months at room temperature^29^. Previous clinical studies have shown that AGE reduces the concentrations of serum IL-6 and TNFα^30–32^. Futhermore, several sulfur compounds in AGE exert various pharmacological effects^29,33–37^. *S*-Allylmercaptocysteine (SAMC), one such pharmacologically active sulfur compound in AGE, has been demonstrated to suppress LPS-induced inflammation via the decreased phosphorylation of NF-κB p65 in a mouse acute respiratory distress syndrome model^38^ and in human gingival epithelial cells^39^. However, the mechanisms underlying this anti-inflammatory effect are not fully understood. In the present study, we found that SAMC inhibited LPS-induced activation of the TLR4 signaling pathway in the HC11 mouse mammary epithelial cell line by increasing the expression of MyD88 short form (MyD88-S), a splice variant of MyD88. Our phosphoproteomic analysis also revealed that the SAMC-induced increase in MyD88-S expression was accompanied by altered phosphorylation of RNA splicing-related proteins.

## Results

### SAMC suppressed the induction of pro-inflammatory cytokines and chemokines by LPS in MECs

We first orally administered AGE or distilled water to mice during pregnancy until 3 days after parturition and then injected LPS into one side of the 4th gland (Supplementary Fig. S1). We performed transcriptome analysis of LPS-treated glands from AGE- or distilled water-treated animals 12 h after LPS injection. The results showed that AGE suppressed the expression of 1,062 of the 2,423 genes that were upregulated by LPS treatment (Supplementary Fig. S1). The gene ontology (GO) biological process and pathway analyses of these inhibited genes revealed that the top enriched processes and pathways included the immune response and signal transduction pathways (Supplementary Fig. S1).

**Fig. 1:**
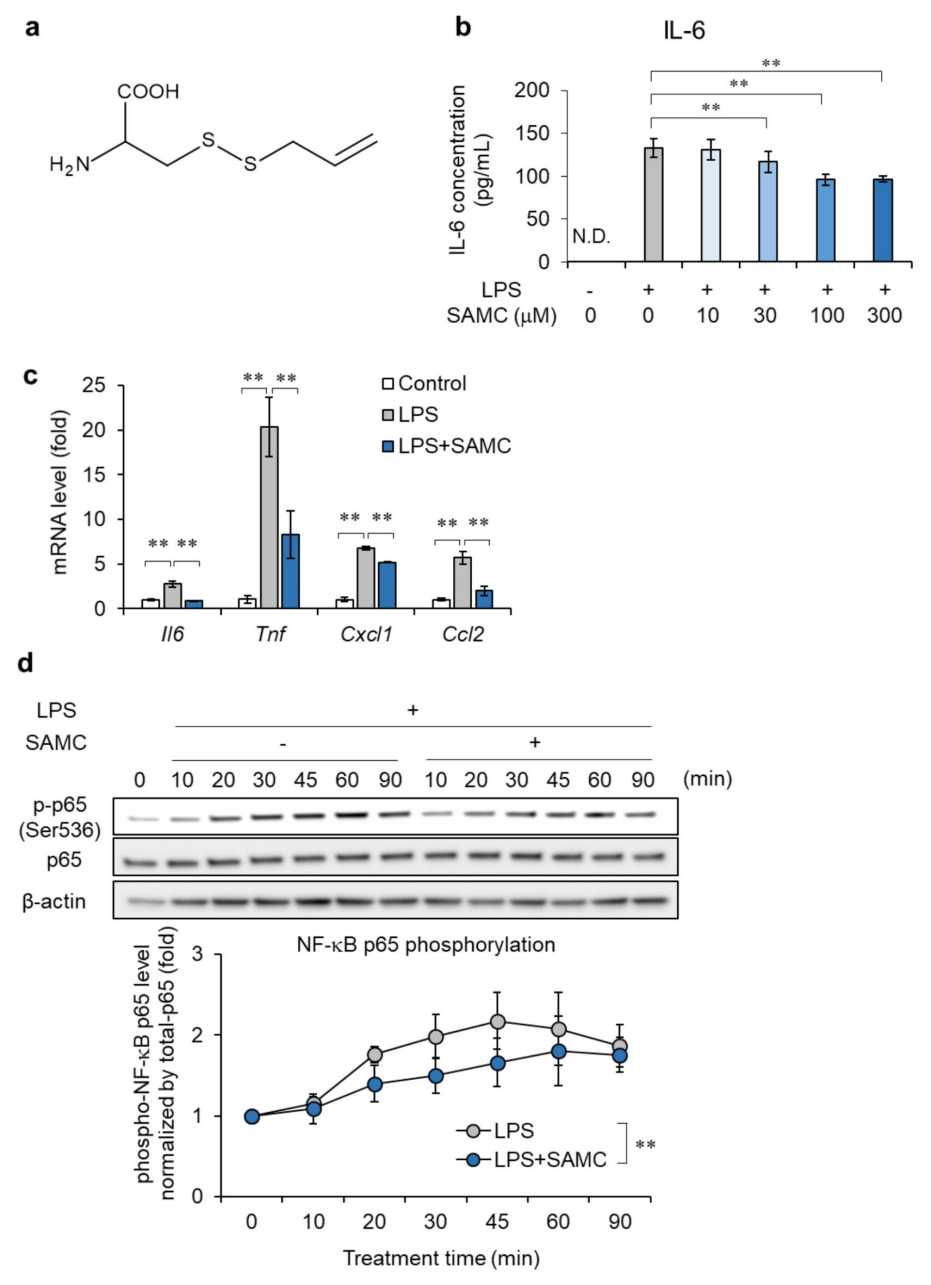
Effect of S-allylmercaptocysteine (SAMC) on the induction of pro-inflammatory cytokines and chemokines and activation of NF-κB by lipopolysaccharide (LPS). (**a**) The chemical structure of SAMC. (**b**) IL-6 secretions in cultured HC11 cells treated with SAMC (10-300 μM) and LPS (100 ng/mL) for 24 h were measured by ELISA. n = 4. (**c**) mRNA expression of pro-inflammatory cytokines (Il6 and Tnfa) and chemokines (Cxcl1 and Ccl2) in HC11 cells treated with LPS and SAMC (300 μM) for 1 h was examined by real-time quantitative PCR. n = 4-5. (**d**) Phosphorylation level of NF-κB p65 were examined by immunoblotting in HC11 cells upon LPS stimulation (1 μg/mL) for 10-90 min and SAMC treatment for 10-90 min. Data are shown as mean±SD, n = 3. ** denotes significant difference (p < 0.01). N.D. means not detected.

Next, we performed experiments to identify the active components of AGE using the HC11 mouse MEC line. We focused on SAMC (Fig. 1a), a sulfur compound found in AGE, because it has previously been demonstrated to have anti-inflammatory effects^38,39^. We performed transcriptome analysis of HC11 cells treated with LPS, with or without SAMC (Supplementary Fig. S2). The results showed that the transcriptomes of cells treated with LPS and SAMC resembled those of control cells (Supplementary Fig. S2). GO biological process and pathway analysis of genes that were induced by LPS but inhibited by SAMC showed that the immune response and signal transduction pathways were enriched by SAMC treatment (Supplementary Fig. S2). These results are consistent with the findings in mammary gland tissues after AGE treatment, suggesting that, as an active component of AGE, SAMC exerts anti-inflammatory effects by inhibiting the LPS-induced TLR4 signaling pathway. We also confirmed that SAMC significantly suppressed the expression of the pro-inflammatory cytokine *Il6* in LPS-treated cells (Supplementary Fig. S2).

Next, we studied the effect of SAMC on the TLR4 signaling pathway in HC11 cells after stimulation with a high concentration of LPS (100 ng/mL) to amplify signal transduction. LPS-induced IL-6 production was reduced by SAMC treatment in a concentration-dependent manner (up to 27.5% reduction) (Fig. 1b). Additionally, SAMC (300 µM) significantly suppressed the mRNA levels of *Il6*, *Tnf*, *Cxcl1*, and *Ccl2* by 68.0%, 59.4%, 23.4%, and 64.6%, respectively, in HC11 cells treated with LPS for 1 h (Fig. 1c). Furthermore, SAMC inhibited the phosphorylation (Fig. 1d and Supplementary Fig. S3) and nuclear translocation (Supplementary Fig. S4) of NF-κB p65 induced by LPS. Therefore, SAMC suppressed LPS-induced TLR4 signal transduction and consequently reduced the production and secretion of pro-inflammatory cytokines.

### Phosphoproteomic analysis revealed that SAMC inhibits LPS-induced global phosphorylation in HC11 cells

**Fig. 2:**
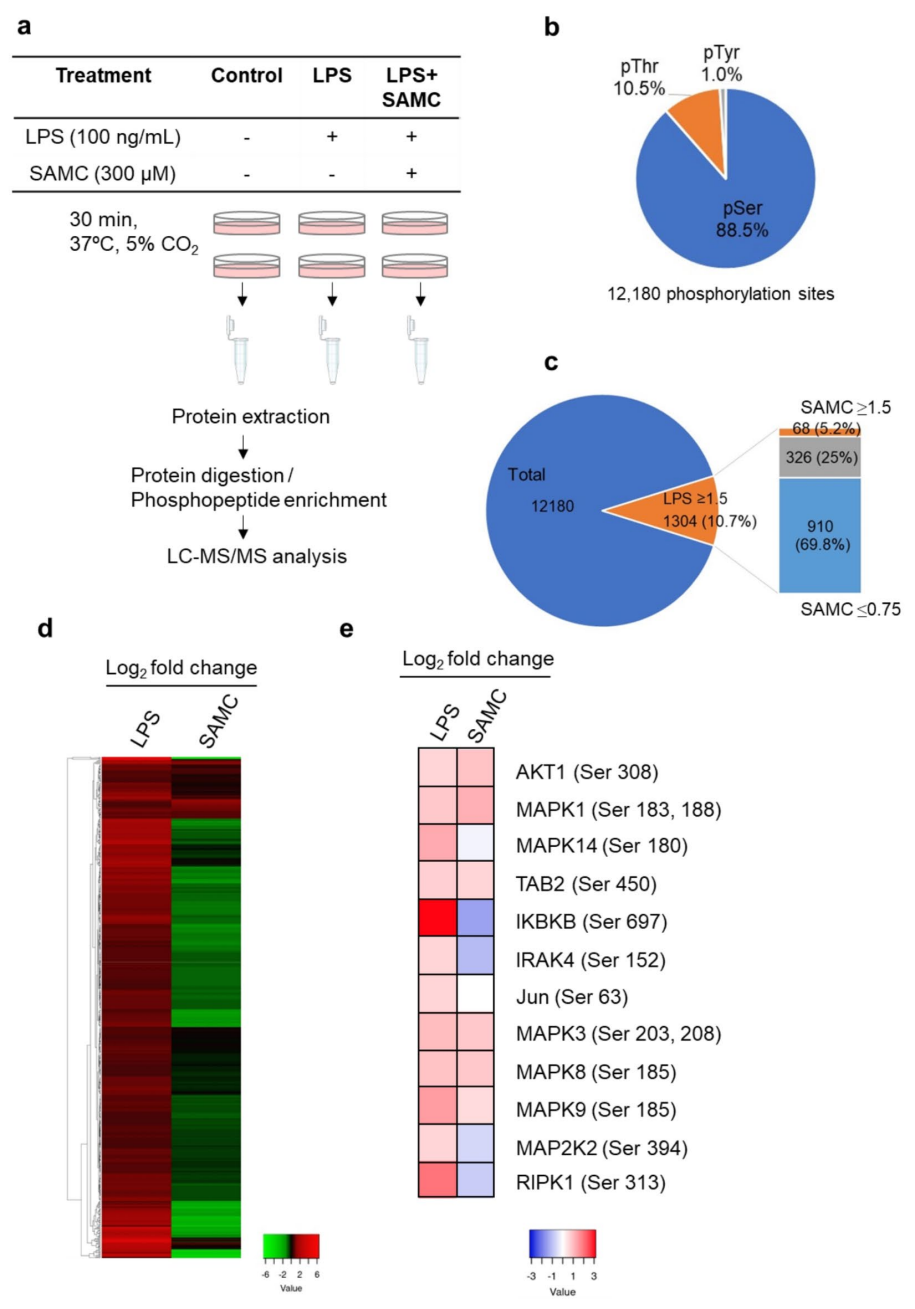
Effects of S-allylmercaptocysteine (SAMC) on phosphoproteome in HC11 cells treated with lipopolysaccharide (LPS). (**a**) Workflow of phosphoproteomic analysis. (**b**) The proportion of phosphorylated amino acid residues in detected phosphopeptides (pSer = phosphorylated serine, pThr = phosphorylated threonine, pTyr = phosphorylated tyrosine). (**c**) The proportion of phosphopeptides increased by LPS stimulation (≥ 1.5-fold, 10.7%) and the proportions of these phosphopeptides increased (≥ 1.5-fold, 5.2%) or decreased (≤ 0.75-fold, 69.8%) by SAMC treatment. (**d**, **e**) The heat map of the phosphopeptides affected by LPS and SAMC in Fig. 2c (**d**) and the phosphorylation level of protein kinases in the TLR signaling pathway (**e**). LPS and SAMC columns show the log2 fold changes of the phosphopeptide abundance in LPS-treated cells vs. control and in LPS- and SAMC- treated cells vs. LPS alone-treated cells, respectively.

Global phosphoproteomic analysis using data-independent acquisition (DIA)-MS was performed to determine whether SAMC altered protein phosphorylation in LPS-treated HC11 cells. The complete workflow is shown in Fig. 2a. No differences in retention time were observed in the total ion current chromatograms obtained from DIA-MS analysis of the phospho-peptide extracts treated with LPS or LPS + SAMC (Supplementary Fig. S5). We identified 12,180 phosphorylation sites across 3,428 proteins. As shown in Fig. 2b, the phosphorylation sites were predominantly on serine residues (88.5%), followed by threonine residues (10.5%), with tyrosine phosphorylation being the lowest (1%). After LPS treatment, the phosphorylation level was increased at 1,304 sites (≥ 1.5-fold change vs. the control), whereas concomitant treatment with SAMC decreased the phosphorylation level at 910 sites (approximately 70%, ≤ 0.75-fold change vs. LPS alone) (Fig. 2c and d).

We also performed WikiPathway analysis using the Database for Annotation, Visualization, and Integrated Discovery (DAVID, ver. 2021) to identify the phosphoproteins in the TLR4 signaling pathway affected by SAMC. Upon LPS stimulation, twelve phosphoproteins with a 1.5-fold increase in the phosphorylation level were identified, whereas SAMC decreased the phosphorylation levels of four of these proteins, including IRAK4 (53.2% decrease) and IKBKB (63.5% decrease) (Fig. 2e and Supplementary Fig. S6). These findings suggest that SAMC affects the phosphorylation status of TLR4 downstream molecules, resulting in the downregulation of the TLR4 signaling pathway.

### SAMC inhibits mRNA splicing by reducing the phosphorylation of U2 snRNP-related proteins

**Fig. 3:**
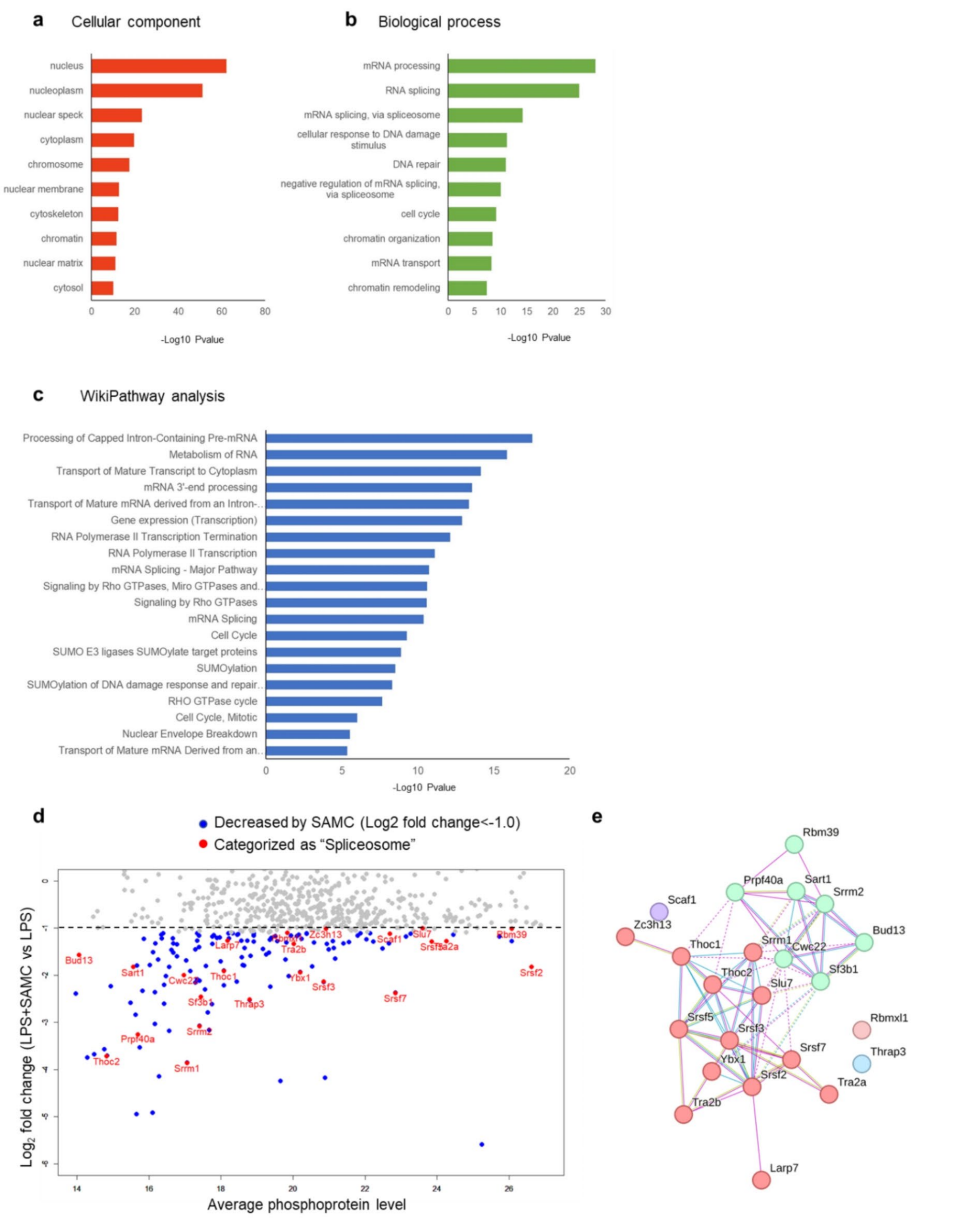
*S*-Allylmercaptocysteine (SAMC) down-regulates the phosphorylation of mRNA splicing-related proteins. (**a**-**c**) Gene ontology term annotation for cellular component (**a**) and biological process (**b**) and Wikipathway analysis (**c**) of the phosphoproteins increased by lipopolysaccharide (LPS) treatment (≥ 1.5-fold vs. control) and decreased by SAMC treatment (≤ 0.75-fold vs. LPS group). (**d**) MA plot of the phosphoproteins downregulated by SAMC. (**e**) Protein-protein interactions among the spliceosome-related proteins in (**d**) were represented by Markov Cluster Algorithm clustering performed by STRING database. The proteins associated with U2 snRNP and other spliceosomes excepting U2 snRNP were shown as blue and red spheres, respectively.

Next, we performed GO term enrichment analysis using DAVID on phosphoproteins upregulated by LPS but downregulated by SAMC. The GO cellular component analysis revealed that several phosphoproteins downregulated by SAMC (≤ 0.75-fold vs. LPS alone) were nuclear components, suggesting the action of SAMC within the nuclei (Fig. 3a). GO biological process analysis of these proteins showed that the processes involved in mRNA processing and splicing were downregulated by SAMC treatment (Fig. 3b). These results are consistent with those of the WikiPathway analysis, suggesting that SAMC-downregulated phosphoproteins are associated with mRNA processing and splicing pathways (Fig. 3c). LPS stimulation also altered these mRNA splicing-related pathways compared to control cells (Supplementary Fig. S7), suggesting that SAMC suppressed LPS-induced changes in mRNA splicing. The differences in phosphoprotein levels in LPS + SAMC-treated cells compared to those in cells treated with LPS alone (≥ 1.5-fold vs. control) are shown in the MA plot in Fig. 3d. The 23 mRNA splicing-related proteins whose phosphorylation levels were reduced by SAMC treatment (≤ 0.5-fold vs. LPS) are presented as red symbols in the MA plot.

We performed STRING analysis of the spliceosome-related proteins represented in the MA plot. The phosphoproteins decreased by SAMC treatment were associated with U2 snRNP and other spliceosomal components and were organized using physical subnetwork and Markov Cluster Algorithm clustering (Fig. 3e). U2 snRNP-related proteins, including splicing factor 3B subunit 1 (SF3B1), accounted for 30.4% of the phosphoproteins linked to mRNA splicing that were decreased by SAMC treatment. These results suggested that SAMC regulates mRNA splicing by reducing the phosphorylation of U2 snRNP components.

### SAMC inhibited the phosphorylation of various kinases in HC11 cells

**Fig. 4:**
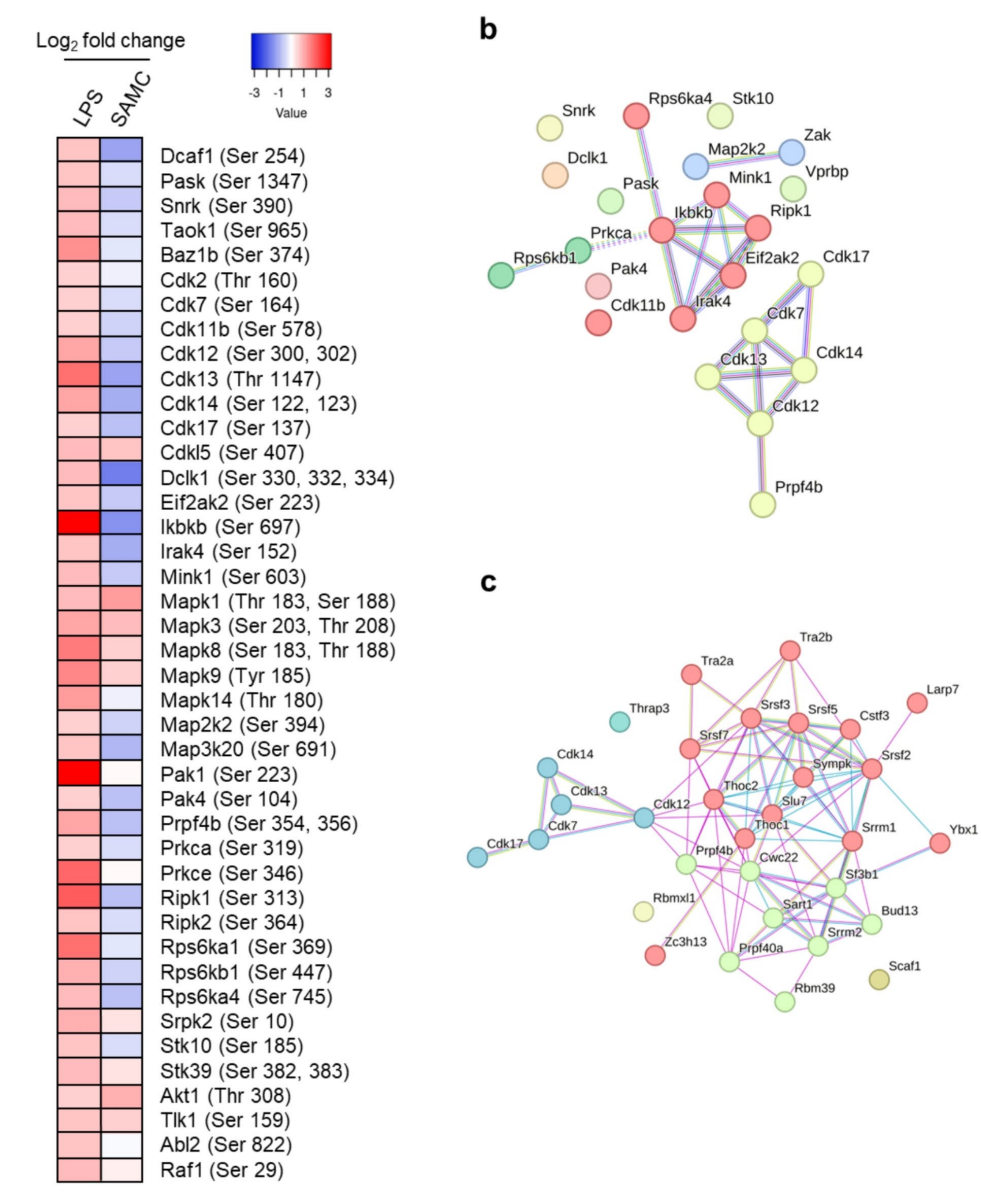
*S*-Allylmercaptocysteine (SAMC) down-regulates the phosphorylation of various kinases in HC11 cells. (**a**) The heatmap of phosphorylation of kinases increased by lipopolysaccharide (LPS) (≥ 1.5-fold). LPS and SAMC columns showed the log_2_ fold changes of the phosphopeptide abundance in LPS vs. control and SAMC vs. LPS, respectively. (**b**) Protein-protein interactions among the kinases increased by LPS (≥ 1.5-fold) and decreased by SAMC (≤ 0.75-fold) represented by Markov Cluster Algorithm (MCL) clustering. TLR-related kinases are shown as red spheres, and CDK family is shown as yellow spheres. (**c**) Protein-protein interactions among the spliceosome-related proteins in Fig. 3f and CDKs family in Fig. 4b represented by MCL clustering. The proteins associated with U2 snRNP, other spliceosome (except U2 snRNP), and CDKs family are shown as green, red, and blue spheres, respectively.

Because LPS has been shown to activate several kinases in previous studies^40,41^, we analyzed the phosphorylation status of these kinases after LPS and SAMC treatment in HC11 cells. The phosphorylation of 42 kinases was up-regulated in response to LPS stimulation (≥ 1.5-fold vs. the control). Among them, SAMC treatment reduced the phosphorylation of 23 proteins (≤ 0.75-fold) (Fig. 4a). Furthermore, STRING analysis revealed that SAMC suppressed the phosphorylation of kinases involved in the TLR4 signaling pathway as well as the CDK family (Fig. 4b). Because previous studies have revealed that CDKs regulate mRNA splicing by activating splicing-related proteins^42^, we performed further STRING analysis of the proteins downregulated by SAMC and identified CDK12 as the kinase linking CDKs and spliceosome-related proteins (Fig. 4c). These results suggest that SAMC suppresses mRNA splicing by inhibiting the activity of CDK family proteins, especially CDK12.

### SAMC increased the expression of Myd88s mRNA in HC11 cells

**Fig. 5:**
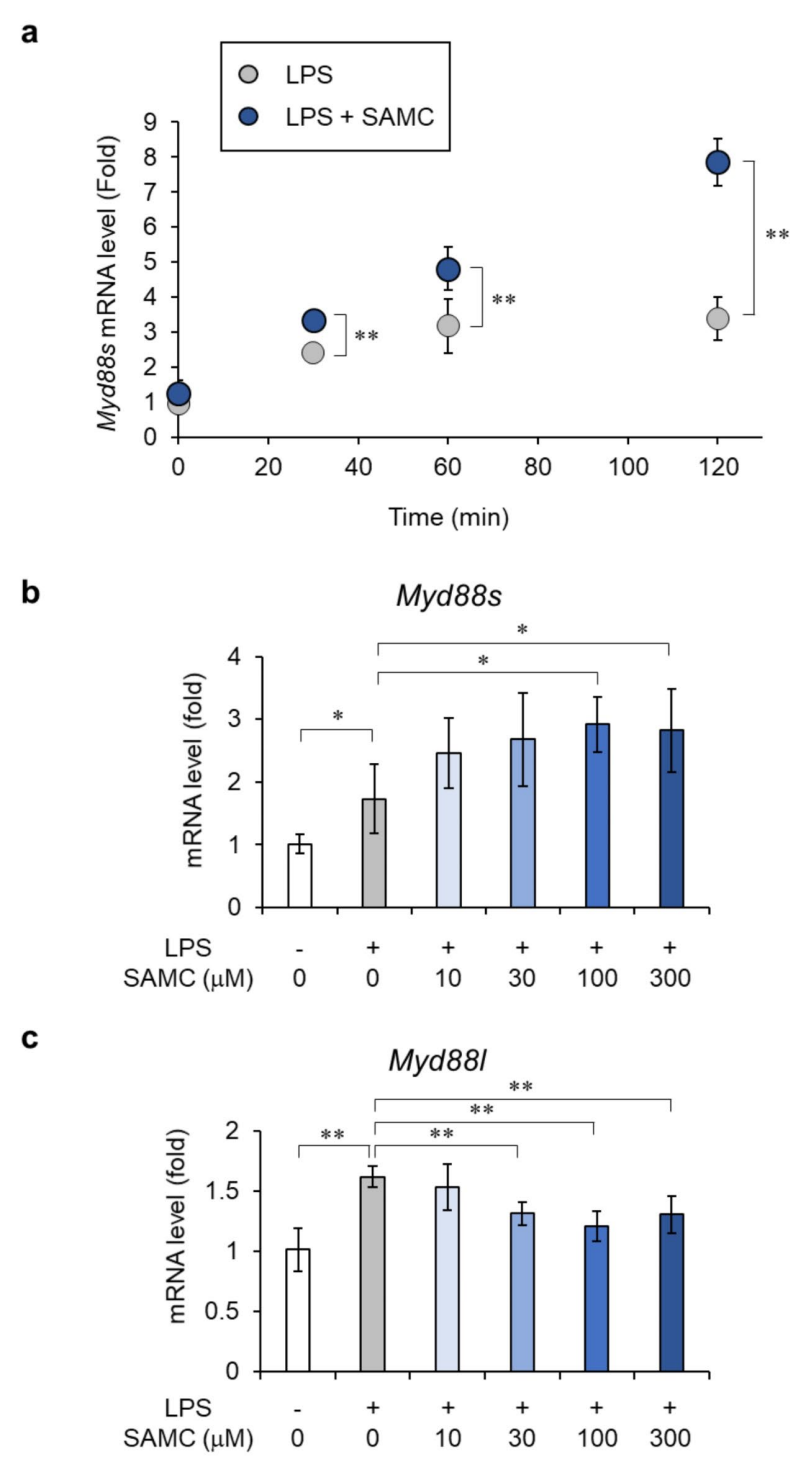
*S*-Allylmercaptocysteine (SAMC) induces the gene expression of MyD88-S. (**a**) Real-time qPCR analysis of the effect of SAMC (300 µM) on mRNA expression of *Myd88s* in HC11 cells upon lipopolysaccharide (LPS) stimulation (100 ng/mL) for 30–120 min. (**b**, **c**) Real-time qPCR analysis of the concentration-dependent effect of SAMC (10–300 µM) on mRNA expression of *Myd88s* (**b**) and canonical *Myd88l* (**c**) in HC11 cells treated with LPS for 2 h. Data are shown as mean ± SD, *n* = 4–5. ** denotes significant difference (*p* < 0.01) and * denotes significant difference (*p* < 0.05).

The inhibition of U2 snRNPs, including SF3A and SF3B, has been reported to increase the expression of MyD88 short form (MyD88-S), an inhibitory splicing variant in the TLR signaling pathway, by regulating MyD88 alternative splicing^43^. Because SAMC inhibited the phosphorylation of U2 snRNP component proteins, including SF3B1 (Fig. 3e), we examined the effect of SAMC on *Myd88s* mRNA levels. As shown in Fig. 5a, SAMC increased *Myd88s* mRNA levels by 2.3-fold at 120 min after treatment compared to LPS treatment alone, and this effect was concentration-dependent (Fig. 5b). In contrast, SAMC reduced the mRNA levels of the MyD88 long form (*Myd88l*), which activates innate immunity by transducing the TLR signaling pathway (Fig. 5c). SAMC treatment increased the ratio of *Myd88s* to *Myd88l* mRNA expression (Supplementary Fig. S8). These results suggest that SAMC suppresses the activation of TLR4 signaling, at least partly because of the increased production of MyD88-S, which is possibly induced by changes in the phosphorylation state of U2 snRNP component proteins.

## Discussion

Mastitis is an inflammatory disease caused by the bacterial infection of the mammary tissues^1^. *E. coli* is a major gram-negative mastitis pathogen that contains LPS, and its infection of the mammary gland can cause substantial damage to mammary tissues, leading to reduced milk yield^44^. In the early stages of mastitis, the inflammatory response of epithelial cells triggers the production of pro-inflammatory cytokines and the infiltration of immune cells into the alveoli of the mammary gland. Inhibiting the inflammatory response in the mammary epithelium is crucial for managing mastitis^8^. In this study, we found that SAMC suppressed the secretion of IL-6 as well as *Il6*, *Tnf*, *Cxcl1*, and *Ccl2* mRNA levels induced by LPS in HC11 cells (Fig. 1c), suggesting that SAMC is a key component of AGE that modulates the immune response in an in vivo mastitis model (Supplementary Fig. S1). LPS, a well-characterized MAMP in *E. coli*, is typically used to induce inflammatory reactions similar to mastitis, including pro-inflammatory cytokine production and immune cell infiltration, in in vivo and in vitro models^45,46^. Previous studies have shown that SAMC inhibits LPS-activated pro-inflammatory pathways and the production of cytokines, including IL-6, in vivo^38^ and in vitro^39^. However, the mechanisms underlying this activity are not fully understood. Garlic-derived sulfur-containing compounds, such as diallyl trisulfide and *S*-propargylcysteine, have been reported as potential donors of hydrogen sulfide (H_2_S) in the presence of reduced glutathione^47–49^. H_2_S exerts anti-inflammatory effects during bacterial infections by regulating protein function via cysteine persulfidation^50,51^. Although the capacity of SAMC to act as an H_2_S donor has not yet been studied, the allyl substituent, which is present in SAMC, undergoes nucleophilic substitution by GSH at its α-carbon to form an allyl perthiol and a thiol/disulfide exchange to generate H_2_S^50^. Thus, SAMC may exert its anti-inflammatory effects by forming allyl perthiols and producing H_2_S. Further studies are required to determine the involvement of H_2_S in the anti-inflammatory effects of SAMC.

TLR4 deficiency has been demonstrated to attenuate inflammatory cytokine production and mammary tissue damage in a mouse model of LPS-induced mastitis^13,52^. In this present study, we found that SAMC inhibited the TLR4 signaling pathway by increasing the production of MyD88-S, an alternatively spliced form of MyD88, by suppressing pre-mRNA splicing. Negative feedback loops in TLR4 signaling terminate inflammation, one of which is mediated by alternative splicing, which produces spliced forms encoding negative regulators of TLR4 signaling, such as MyD88-S^53–55^. Because of the lack of an intermediate domain caused by exon 2 skipping, MyD88-S cannot bind IRAK4, resulting in its inability to recruit IRAK4 to MyD88 on the plasma membrane. Consequently, this inhibits the activation of the downstream signaling pathway of TLR4, including NF-κB^56–58^. SAMC inhibited the LPS-induced phosphorylation of IRAK4 and IKKβ (Fig. 2e), suggesting that it can inhibit the downstream signaling pathway of IRAK4 by inducing MyD88-S expression, thus promoting a splicing-mediated negative feedback loop following LPS stimulation.

mRNA splicing is initiated by the binding of U1 snRNP to the 5′ end of the intron and their interactions with U2 snRNP at the branch point within the intron, via SF3A1 and SF3B1^59,60^. In addition to canonical splicing, Myd88 pre-mRNA undergoes alternative splicing owing to a sequence in intron 1 that is relatively prone to splice switching. The polypyrimidine tract sequence of intron 1 of *Myd88* pre-mRNA binds to the spliceosome component U2 small nuclear ribonucleoprotein auxiliary factor (U2AF) with moderate strength owing to the presence of a significant number of thymidine and cytidine residues. However, the branch point sequence of *Myd88* intron 1 deviates profoundly from the expected sequence, indicating weak binding to U2 snRNP at this branch point^61^. Furthermore, several reports have suggested that knockdown of SF3A1, SF3B1, U2AF, or SRSF2 inhibits LPS-induced IL-6 production by upregulating MyD88-S^43,62,63^. Accordingly, exon 2 skipping of *Myd88* pre-mRNA may be caused by weakening the interaction between intron 1 of *Myd88* pre-mRNA and U2 snRNP, rendering intron 1 unrecognizable. Our results demonstrate that SAMC downregulates the phosphorylation levels of several spliceosomal components, especially U2 snRNP. Additionally, SAMC inhibited the phosphorylation of various CDKs, including CDK12/13, which may play a role in regulating mRNA splicing and the phosphorylation of spliceosomal components.

In conclusion, our phosphoproteomic analysis showed that SAMC induces the splicing of MyD88 toward its short form by inhibiting the phosphorylation of spliceosomal components, which may suppress LPS-induced IL-6 production in HC11 MECs. However, the effect of SAMC was only examined under inflammatory conditions with LPS stimulation, which is a limitation of this study that should be addressed in future research. Nonetheless, because SAMC did not affect cell survival under non-inflammatory conditions (Supplementary Fig. S9), it may fine-tune the immune response without adversely affecting the mRNA splicing of other genes. In addition, previous studies on various disease models and cultured cells^38,39,64^ have suggested that the anti-inflammatory effect of SAMC is generally applicable to other tissues and cell types. Therefore, SAMC may ameliorate various inflammatory diseases, including mastitis, by modulating immune responses.

## Methods

### Preparation of AGE and SAMC

AGE was manufactured^36^ and SAMC was synthesized and purified^65^ as previously described. The chemical structure of SAMC was determined by liquid chromatography mass spectrometry (LC-MS) using an Ultimate 3000 (Dionex, Sunnyvale, CA, USA) and a Q-Exactive (Thermo Fisher Scientific, Waltham, MA, USA), and by a VNMRS-500 NMR spectrometer (Varian, Palo Alto, CA, USA).

### Animals and treatment

Twenty four 8-week old female BALB/c mice were purchased from the Jackson Laboratory (Bar Harbor, ME, USA) and used in the present study. All animal procedures were approved by the University of Vermont Institutional Animal Care and Use Committee (Protocol #18–030) and performed in accordance with the US federal Animal Welfare Act and the Public Health Service Policy on Humane Care and Use of Laboratory Animals. All procedures were also conducted in accordance with ARRIVE guidelines. The mice were fed ad libitum and housed at the University of Vermont Small Animal Facility in breeding cages in a room with a temperature of 25 °C, humidity of 45%, and 12-h dark-light cycles. The mice were bred and orally administered with AGE (2 g/10 mL/kg body weight; AGE group, *n* = 13) or distilled water (Control group, *n* = 10) once a day from the first day of pregnancy until 3 days after parturition (approximately 22 days). On day 3 of lactation, the mice were anesthetized with 4% isoflurane, and the 4th mammary gland on one side was injected with either 50 µL of 0.4 mg/mL LPS (*E. coli* 055: B5, Sigma-Aldrich, St. Louis, MO, USA) through the teat meatus, or 50 µL of PBS for normal control tissue through the teat meatus using 30G insulin needles (Becton, Dickinson & Company, Franklin Lakes, NJ, USA). After 12 h, the animal was then euthanized by exsanguination under anesthesia with 4% isoflurane and the LPS- and PBS-infused mammary glands from each mouse were collected, frozen in liquid nitrogen, and stored at -80 °C.

### Cell culture and treatment

Cells of mouse mammary epithelial cell line HC11 (CRL-3062, ATCC, Manassas, VA, USA) were cultured in RPMI-1640 medium (Thermo Fisher Scientific or Wako Pure Chemical Industries, Osaka, Japan) containing 10% fetal bovine serum (FBS, Thermo Fisher Scientific or Biosera, Cholet, France), 1% antibiotic and antimycotic (Thermo Fisher Scientific) or 1% penicillin-streptomycin (Wako Pure Chemical Industries), 5 µg/mL insulin (Sigma-Aldrich), and 0.1 µM epidermal growth factor (Sigma-Aldrich) in 5% CO_2_ at 37ºC. For RNA sequencing, the cells were pre-treated with or without 100 µM SAMC in RPMI-1640 medium containing 2% charcoal-stripped horse serum (Valley Biomedical Products & Services, Winchester, VA, USA), 1% antibiotic and antimycotic, and 5 µg/mL insulin for 24 h, then treated with 100 µM SAMC, 0.5 ng/mL LPS in the above-mentioned medium with 0.1 µM dexamethasone (Sigma-Aldrich) and 5 µg/mL prolactin (Sigma-Aldrich) for 3 h. For the measurement of IL-6 concentration, cell viability assay, reverse transcription-quantitative polymerase chain reaction (RT-qPCR), western blotting, and phosphoproteomic analysis, the cells were pre-cultured overnight in the low (2%)-FBS medium containing 1% penicillin-streptomycin prior to all treatments.

### RNA sequencing

A total of 30 mg of frozen mammary gland tissue was ground by a mortar and pestle chilled with liquid nitrogen and subsequently placed in 600 µL of lysis buffer (Qiagen, Hulsterweg, Netherlands). Total RNA was extracted from the LPS-infused and PBS-infused mammary glands of five randomly selected mice (*n* = 5) and from HC11 cells after treatment with LPS (*n* = 4) using the RNeasy Mini Kit (Qiagen) according to the manufacturer’s protocol. The total RNA samples were sent to Novogene (Beijing, China) for library preparation using the NEBNext^®^ UltraTM RNA Library Prep Kit for Illumina^®^ (New England Biolabs, Ipswich, MA, USA) and sequencing. The detailed protocol was described previously^9^.

### Measurement of IL-6 level

After the treatment of the cells with LPS (100 ng/mL) in the presence or absence of SAMC (10–300 µM) for 24 h, the culture medium was collected and frozen at -80ºC until use. The IL-6 content in the medium was measured by an IL-6 Mouse Uncoated ELISA Kit (Thermo Fisher Scientific) according to the manufacturer’s instructions.

### Cell viability assay

Cell viability was assessed using the Cell Counting Kit-8 (Dojindo Molecular Technology, Kumamoto, Japan) according to the manufacturer’s instructions.

### RT-qPCR

After the treatment of the cells with LPS and/or SAMC (10–300 µM) for 30–120 min, total RNA was isolated from cells with TRIzol reagent (Thermo Fisher Scientific). Complementary DNA (cDNA) was synthesized by a PrimeScript RT reagent kit with genomic DNA Eraser (Takara, Shiga, Japan). Synthesized cDNA was amplified with primer pairs using a KAPA SYBR Fast qPCR kit (KAPA Biosystems, Wilmington, MA, USA). Quantitative real-time PCR was performed using CFX96 System (Bio-Rad, Hercules, CA, USA) to determine the relative expression level of target genes to the level of hypoxanthine phosphoribosyltransferase 1 gene (*Hprt*). The relative mRNA level was calculated using the comparative CT (ΔΔCT) method^66^. The primers used are listed in Supplementary Table S1.

### Western blotting

After the treatment of the cells with LPS and/or SAMC for the indicated period, total protein was extracted with RIPA lysis buffer (Merck Millipore, Burlington, MA, USA) containing protease and phosphatase inhibitors (Roche, Basel, Switzerland). The protein amount in the extract was quantified using a BCA Protein Assay Kit (Thermo Fisher Scientific). The protein lysates were boiled in sample buffer solution with 3-mercapto-1,2-propanediol (Wako Pure Chemical Industries) at 95ºC for 5 min. The samples were separated on 4–15% SDS-PAGE gradient gel (Bio-Rad) and transferred to polyvinylidene fluoride membranes (Bio-Rad). The membranes were treated with blocking solution (Nacalai Tesque, Kyoto, Japan) for 10 min at room temperature, followed by incubation with an anti-phosphorylated NF-κB p65 (Ser536) (1:1,000; Cell Signaling Technology, Danvers, MA, USA) or anti-NF-κB p65 (1:1,000; Santa Cruz Biotechnology, Dallas, TX, USA) primary antibody at 4ºC overnight. The membrane was then incubated with an HRP-conjugated secondary antibody (1:4,000, Cell Signaling Technology) for 1 h at room temperature. Immunoreactive proteins were detected and visualized with Immunostar Zeta and LD (Wako Pure Chemical Industries), using a ChemiDoc Touch MP Imaging System (Bio-Rad). Densitometric quantification was performed using Image Lab Software (Bio-Rad). The blots were re-probed with the anti-β-actin antibody (1:4,000; MBL Life Science, Tokyo, Japan), and the abundance of β-actin was used to normalize the abundance of other proteins.

### Phosphoproteomic analysis

After the treatment of the cells with LPS (100 ng/mL) and/or SAMC (300 µM) for 30 min, total protein was extracted with lysis buffer. Two samples of cell lysates from the same treatment group were mixed into a single pooled sample (Fig. 2a). Sample preparation and phosphoproteomic analysis were conducted by Kazusa DNA Research Institute (Chiba, Japan). Briefly, the cell lysates were adjusted to 5 µg/µL with 100 mM Tris-HCl, 0.5% sodium dodecanoate (Sigma-Aldrich) and phosphatase inhibitors. Proteins in the extract were reduced with dithiothreitol, alkylated with iodoacetamide, and digested with trypsin/Lys-C. Phosphopeptides were enriched using a Titansphere Phos-TiO Kit (GL Sciences, Tokyo, Japan) and desalted using a GL-Tip SDB (GL Sciences). The obtained peptides were loaded onto nano-HPLC capillary column (Nikkyo Technos, Tokyo, Japan) with an UltiMate 3000 RSLCnano system (Thermo Fisher Scientific). Peptides eluting from the column were analyzed on a Q Exactive HF-X mass spectrometer (Thermo Fisher Scientific) for DIA-MS analysis. The DIA-MS spectra were acquired in the resolution of 30,000 with stepped normalized collision energies of 22, 26, and 30. The overlapping window patterns at m/z 390 to 1,010 (isolation window width 10 Da) were used. To generate the library for DIA measurements, an aliquot of each phosphopeptide sample was applied to the gas-phase fractionation method. The MS spectra were acquired in the scan range of *m*/*z* 395 to 555, 545 to 705, and 695 to 1,005 at 120,000 resolutions and the resulting fragment spectra were acquired at 60,000 resolutions. DIA-MS files were processed by the Scaffold DIA (Proteome Software, Portland, OR, USA). The setting parameters were as follows: digestion enzyme, trypsin; fixed modification, carbamidomethylation (C); variable modification, phospho (S, T, Y). The peptide identification threshold was a peptide false discovery rate (FDR) < 1%.

### Bioinformatic analysis

RNA sequencing and phosphoproteomic data were visualized as heatmaps created by Heatmapper (http://www.heatmapper.ca/). The differentially expressed genes and abundant phosphoproteins were subjected to functional analysis by DAVID online software^67,68^ and WikiPathways database^69^ (https://www.wikipathways.org/). Protein-protein interactions were represented by a network diagram created by STRING database^70^ (https://string-db.org/) and Cytoscape (https://cytoscape.org/index.html).

### Statistical analysis

Data were presented as the mean ± standard deviation (SD). One-way analysis of variance (ANOVA) followed by Bonferroni’s multiple comparison test was used to evaluate statistical significance among multiple groups within one treatment. Two-way ANOVA followed by Hotelling’s T-square test was performed to analyze statistical significance among groups with two independent valuables (treatment and time) using FreeJSTAT Version 22.0E (M. Sato, Japan). A *p*-value < 0.05 was considered to be statistically significant.

## Electronic supplementary material

Below is the link to the electronic supplementary material.

Supplementary Material 1

## Data Availability

The sequencing data have been deposited in the DDBJ Sequence Read Archive database under the accession code DRA017697. Phosphoroteomic files were deposited at the Proteome Xchange^71^ Consortium via the JPOST partner repository^72^ (https://repository.jpostdb.org) under the identifiers PXD048365 for ProteomeXchange and JPST002450 for jPOST.
