# Supplementary material for: S-allylmercaptocysteine inhibits TLR4-mediated inflammation through enhanced formation of inhibitory MyD88 splice variant in mammary epithelial cells
Source: Sci Rep. 2024 Nov 28;14:29627. doi: 10.1038/s41598-024-81304-2 (PMC11604973; doi:10.1038/s41598-024-81304-2)
Supplement: Supplementary file 1 — Supplementary Material 1 [file 41598_2024_81304_MOESM1_ESM.pdf]

Supplementary Information

S-Allylmercaptocysteine inhibits TLR4-mediated inflammation through enhanced formation of inhibitory MyD88 splice variant in mammary epithelial cells

Miyuki Takashima[1*], Masahiro Kurita[2], Haruhi Terai[2],
Feng-Qi Zhao[3], and Jun-ichiro Suzuki[2]

[1] Drug Discovery Laboratory, Wakunaga Pharmaceutical Co., Ltd., Hiroshima, 1624 Shimokotachi, Koda-cho, Akitakata-shi, Hiroshima, 739-1195, Japan.
[2] Central Research Institute, Wakunaga Pharmaceutical Co., Ltd., Hiroshima, 1624 Shimokotachi, Koda-cho, Akitakata-shi, Hiroshima, 739-1195, Japan.
[3] Department of Animal and Veterinary Sciences, University of Vermont, 102 Terrill, 570 Main Street, Burlington, VT, 05405, USA.

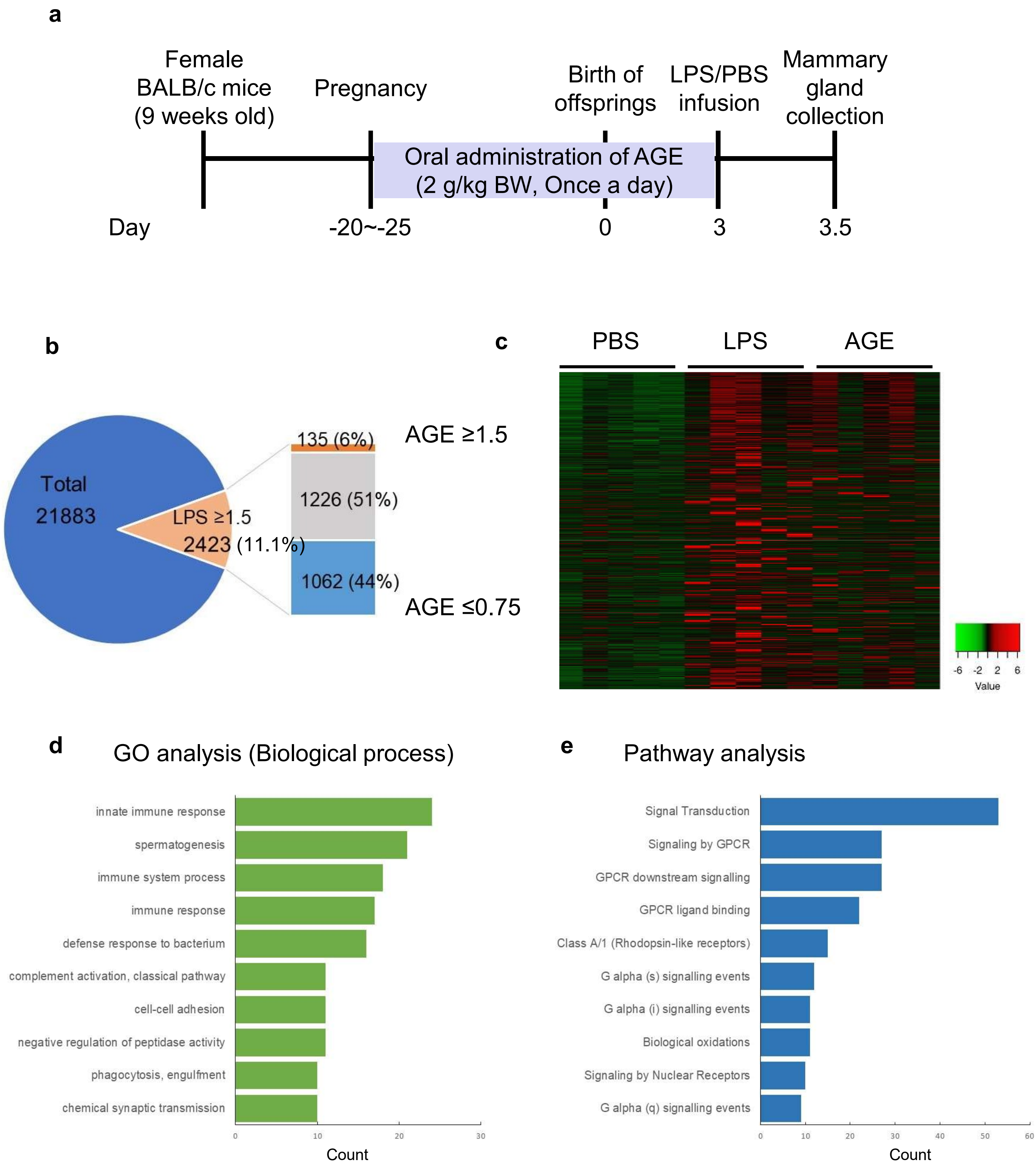

Supplemental Figure S1. Effects of aged garlic extract (AGE) treatment on transcriptome of the mammary glands treated with lipopolysaccharide (LPS) in mice (n= 5). (a) Workflow of animal treatment. (b) The proportion of genes increased by intramammary LPS injection (≥ 1.5-fold vs PBS-injected side, 11.1%) and the proportions of these genes increased (≥ 1.5-fold vs LPS-injected tissues of control mice, 6.0%) or decreased (≤ 0.75-fold, 44.0%) by AGE administration. (c) The heatmap of gene expression increased by LPS (≥ 1.5-fold). PBS and LPS columns show the gene expression in the mammary glands of PBS- and LPS-injected side of control mice, respectively. AGE column shows the gene expression in the LPS-injected mammary glands of mice administered with AGE. (d, e) Gene ontology (GO) term annotation for biological process (d) and pathway analysis (e) of the genes increased by LPS injection (≥ 1.5-fold) and decreased by AGE administration (≤ 0.75-fold).

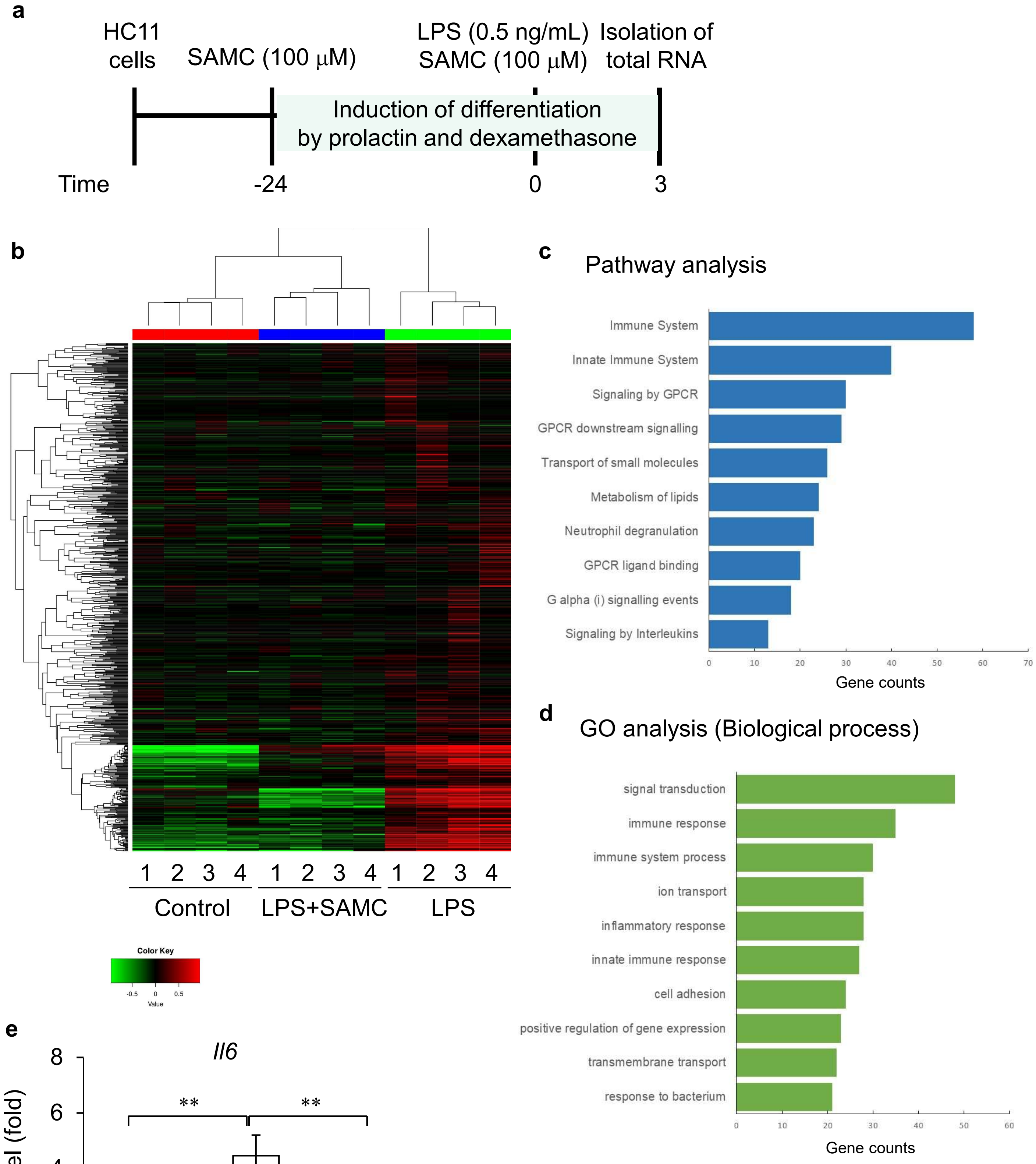

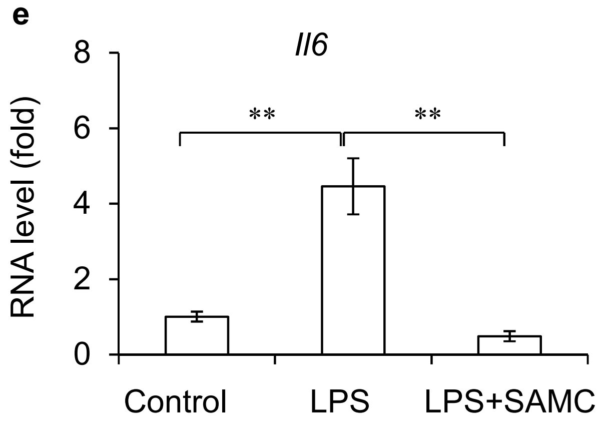

Supplemental Figure S2. Effects of *S*-allylmercaptocysteine (SAMC) on transcriptome and pro-inflammatory cytokine induction in HC11 cells treated with lipopolysaccharide (LPS). (a) Workflow of *in vitro* study using differentiated HC11 cells. (b) The heatmap of gene expression affected by LPS (≥ 1.5-fold). (c, d) Pathway analysis (c) and GO term annotation for biological process (d) of the genes increased by LPS (≥ 1.5-fold) and decreased by SAMC treatment (≤ 0.75-fold). (e) mRNA expression of *Il6* in HC11 cells was examined by real-time quantitative PCR. Data are shown as mean±SD, n = 4-5. ** denotes significant difference ($p < 0.01$).

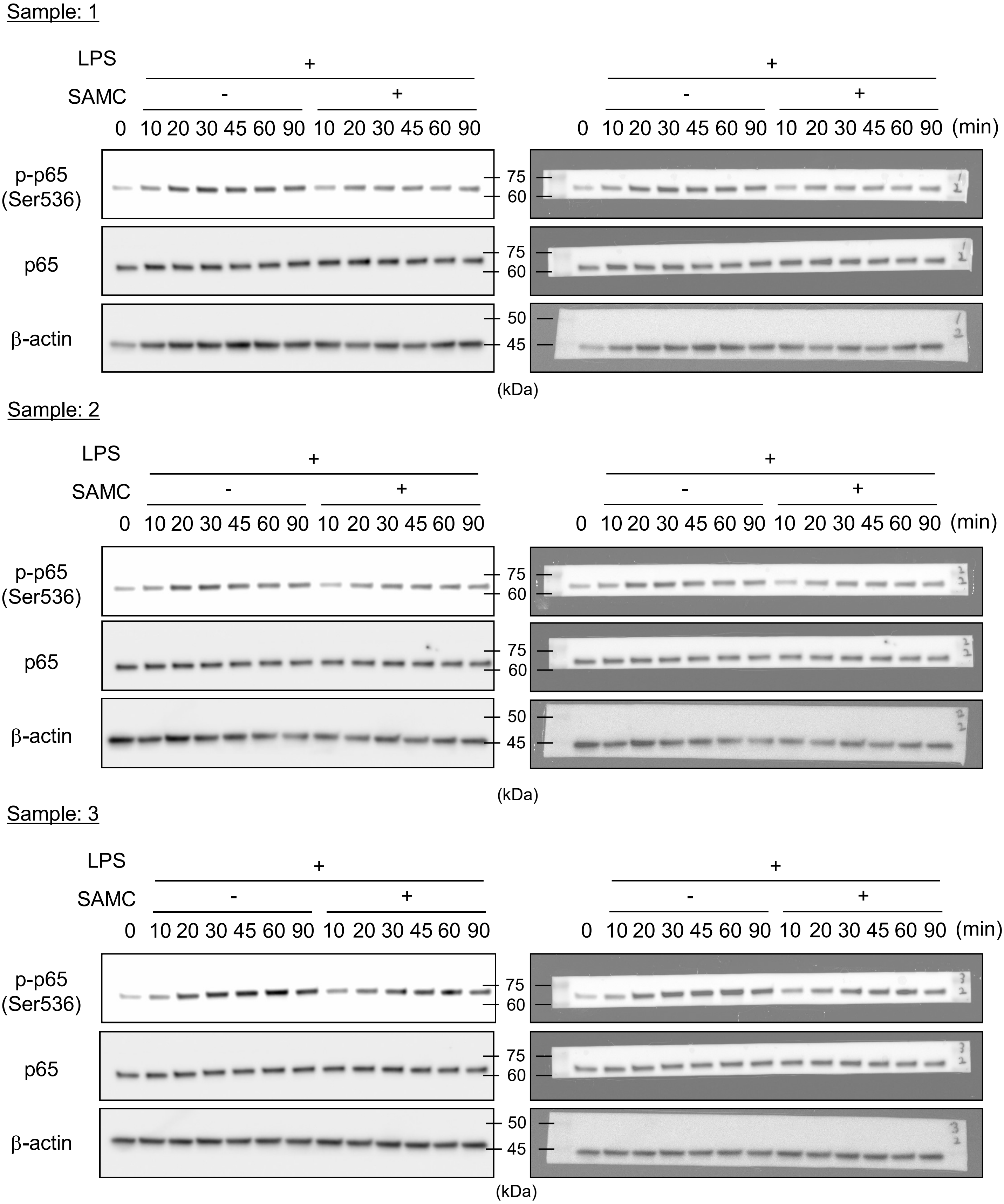

Supplemental Figure S3. Western blots of NF-κB p65 phosphorylation in HC11 cells treated with or without lipopolysaccharide (LPS) and *S*-allylmercaptocysteine (SAMC). Molecular size markers are indicated between the blots used in Fig. 1c. Kda = kilodalton.

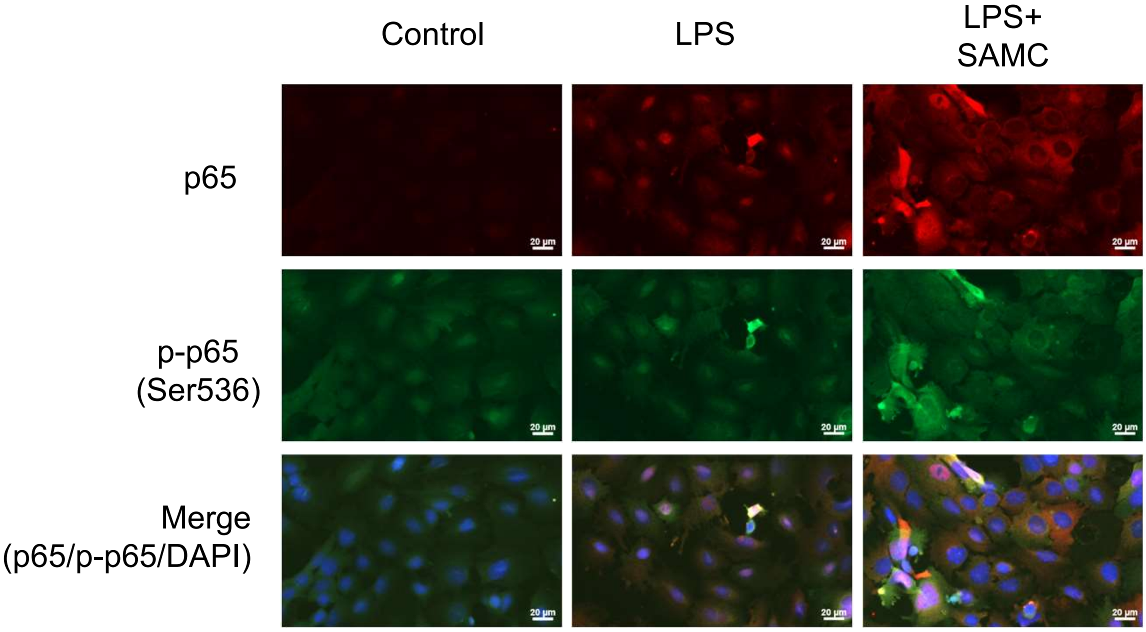

Supplementary Figure S4. Effect of *S*-allylmercaptocysteine (SAMC) on lipopolysaccharide (LPS)-induced activation of NF-κB p65. HC11 cells were immunostained with anti-p65 (red), anti-p-p65 (Ser536) (green) and DAPI (nucleus) (blue) after stimulation with LPS (100 ng/mL) in the presence or absence of SAMC (300 µM) for 1 h. Scale bar, 50 µm.

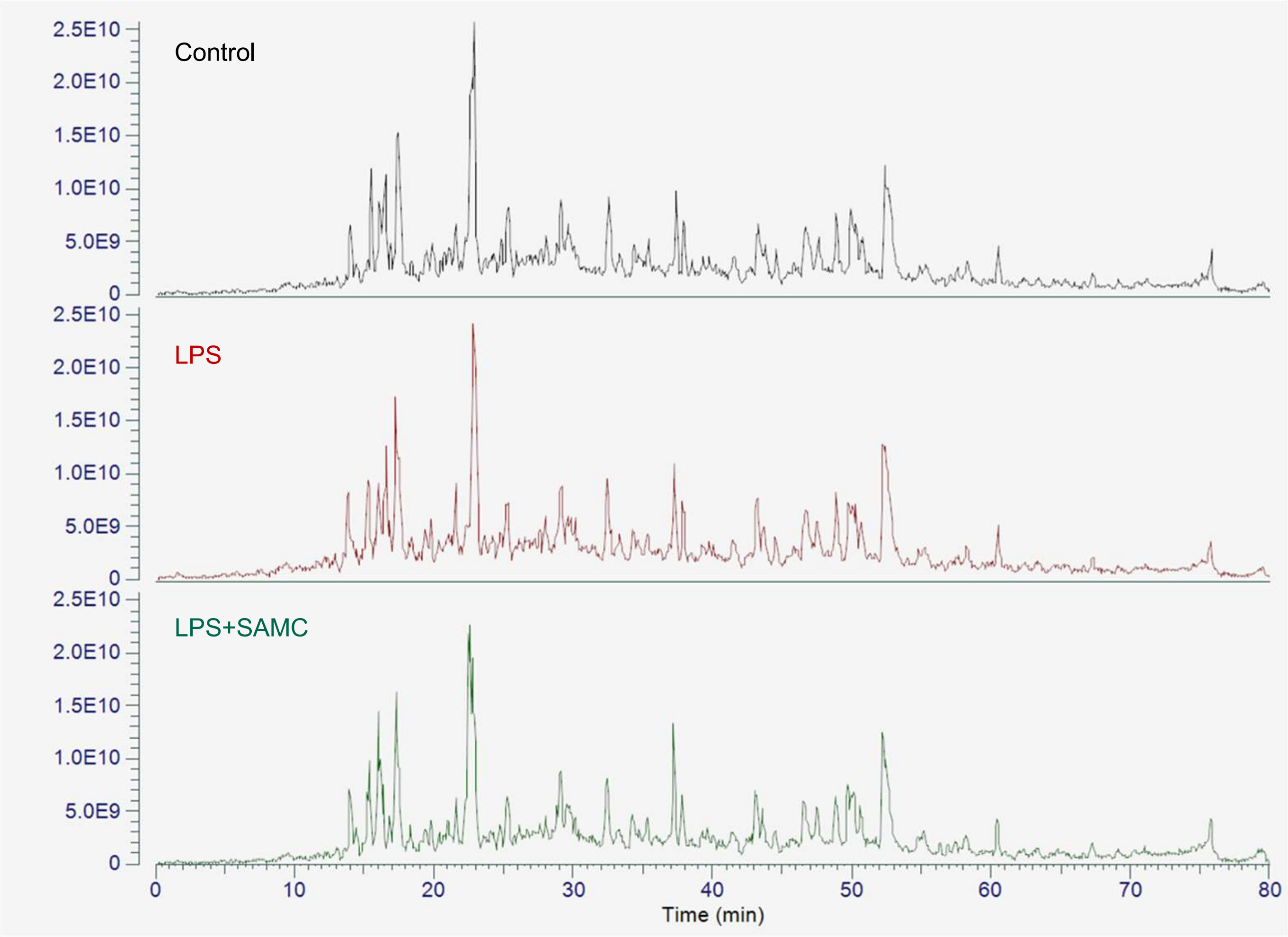

Supplementary Figure S5. Total ion current chromatograms obtained from data-independent acquisition-MS analysis of phospho-peptide extracts.

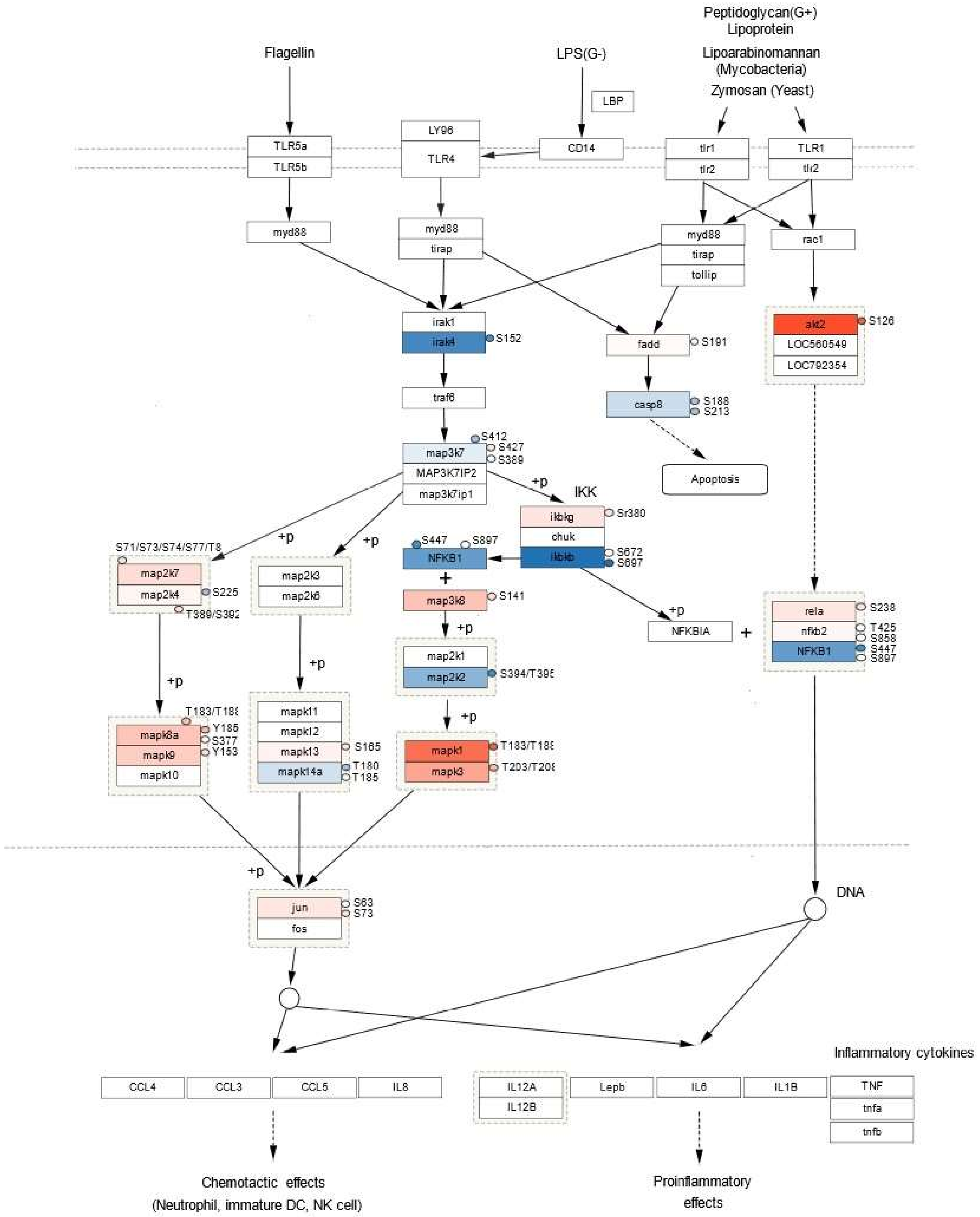

Supplementary Figure S6. The phosphorylation level altered by *S*-allylmercaptocysteine (SAMC) in TLR signaling molecules after lipopolysaccharide (LPS) stimulation. Differentially abundant phosphoproteins in TLR4 pathway between LPS alone- and LPS+SAMC- treated cells were shown in TLR4 signaling pathway map created by Cytoscape. The phosphoproteins increased and decreased by SAMC are labelled with red and blue, respectively.

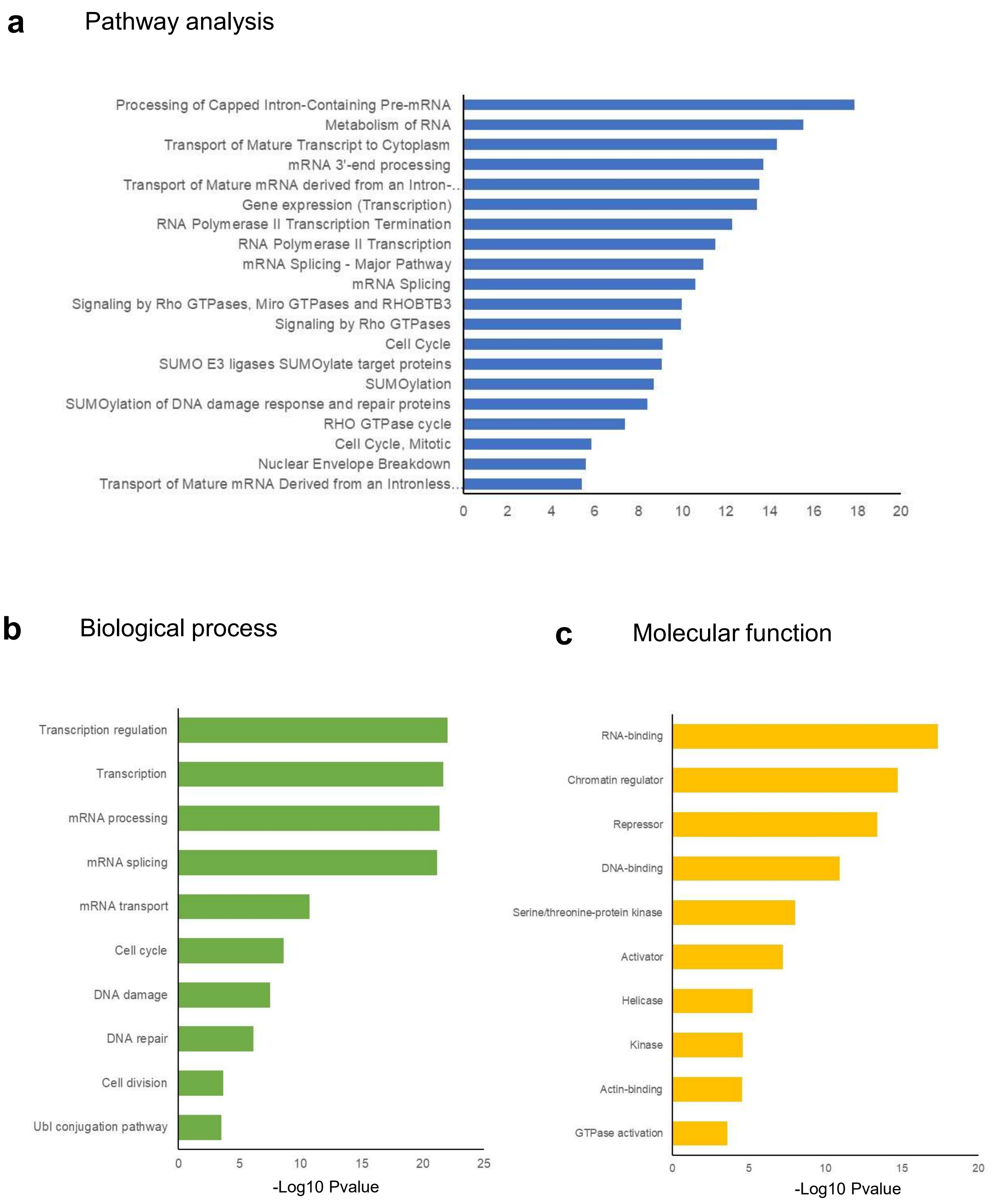

Supplementary Figure S7. Effect of lipopolysaccharide (LPS) on phosphorylation of mRNA splicing-related proteins. Pathway analysis (a), gene ontology term annotation for biological process (b) and molecular function (c) of the genes increased by LPS (≥ 1.5-fold vs. Control).

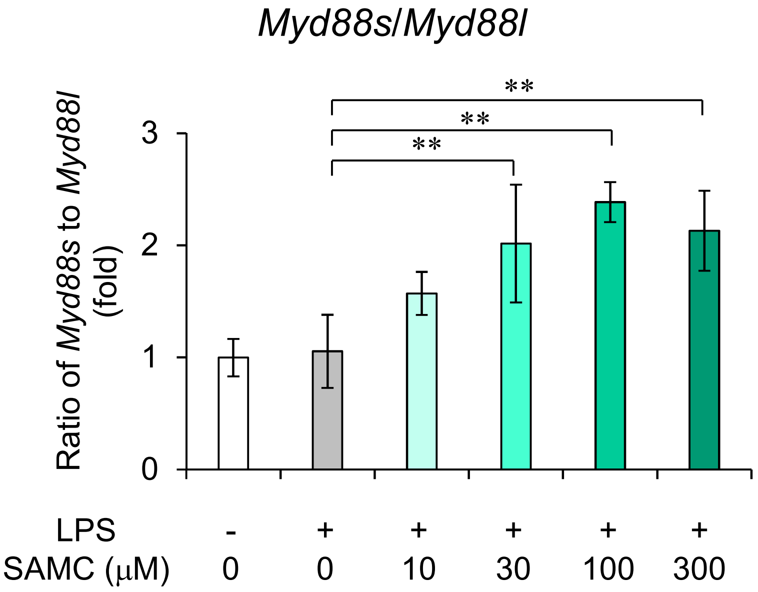

Supplementary Figure S8. *S*-Allylmercaptocysteine (SAMC) increases the mRNA expression ratio of *Myd88s* to canonical *Myd88l*. Real-time qPCR analysis of the concentration-dependent effect of SAMC (10-300 μM) on the mRNA expression ratio of *Myd88s* to canonical *Myd88l* in HC11 cells treated with lipopolysaccharide (LPS) stimulation for 2 h. Data are shown as mean±SD, n = 5. ** denotes significant difference (p<0.01).

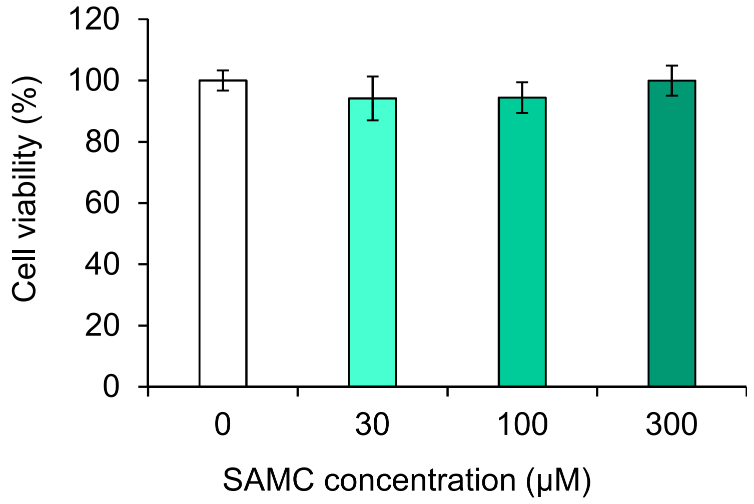

Supplementary Figure S9. *S*-Allylmercaptocysteine (SAMC) has no effect on the viability of HC11 cells. After treatment with SAMC (30-300 μM) for 24 h, the HC11 cell viability was evaluated by WST-8 assay. Data are shown as mean ± SD, n = 5.

Supplementary Table S1. Primer sequences used in this study.

Genes		Primer sequences
Mouse *Il6*	forward	5'-ATGATGCTGGTGACAACCACGG-3'
	reverse	5'-CAGGTCTGTTGGGAGTGGTATCC-3'
Mouse *Tnf*	forward	5'-CTGAACTTCGGGGTGATCGG-3'
	reverse	5'-GGCTTGTCACTCGAATTTTGAGA-3'
Mouse *Cxcl1*	forward	5'-CACTGCACCCAAACCGAAGTC-3'
	reverse	5'-GGGAGCTTCAGGGTCAAGGC-3'
Mouse *Ccl2*	forward	5'-CATCCACGTGTTGGCTCA-3'
	reverse	5'-GATCATCTTGCTGGTGAATGAGT-3'
Mouse *Myd88s*	forward	5'-GGAGCTGAAGTCGCGCATCGGACAAAC-3'
	reverse	5'-GTCTGTTCTAGTTGCCGGATCATCTCCTGCAC-3'
Mouse *MyD88l*	forward	5'-ACCACCCTTGATGACCCCCT-3'
	reverse	5'-GTCACGGTCGGACACACACA-3'
Mouse *Hprt*	forward	5'-GCAGTACAGCCCCAAAATGG-3'
	reverse	5'-TCCAACAAAGTCTGGCCTGT-3'
